# MicroRNAs expression profile in CCR6^+^ regulatory T cells

**DOI:** 10.7717/peerj.575

**Published:** 2014-09-18

**Authors:** Juanjuan Zhao, Yongju Li, Yan Hu, Chao Chen, Ya Zhou, Yijin Tao, Mengmeng Guo, Nalin Qin, Lin Xu

**Affiliations:** 1 Department of Immunology, Zunyi Medical College, Guizhou, China; 2 Department of Medical Physics, Zunyi Medical College, Guizhou, China

**Keywords:** CCR6, miRNAs, Regulatory T cell, Microarray

## Abstract

**Backgroud.** CCR6^+^ CD4^+^ regulatory T cells (CCR6^+^ Tregs), a distinct Tregs subset, played an important role in various immune diseases. Recent evidence showed that microRNAs (miRNAs) are vital regulators in the function of immune cells. However, the potential role of miRNAs in the function of CCR6^+^ Tregs remains largely unknown. In this study, we detected the expression profile of miRNAs in CCR6^+^ Tregs.

**Materials and Methods.** The expression profile of miRNAs as well as genes in CCR6^+^ Tregs or CCR6^-^ Tregs from Balb/c mice were detected by microarray. The signaling pathways were analyzed using the Keggs pathway library.

**Results.** We found that there were 58 miRNAs significantly upregulated and 62 downregulated up to 2 fold in CCR6^+^ Tregs compared with CCR6^-^ Tregs. Moreover, 1,391 genes were observed with 3 fold change and 20 signaling pathways were enriched using the Keggs pathway library.

**Conclusion.** The present data showed CCR6^+^ Tregs expressed specific miRNAs pattern, which provides insight into the role of miRNAs in the biological function of distinct Tregs subsets.

## Introduction

CC chemokine receptor type 6 (CCR6), a family member of chemokine receptor, was widely expressed in various immune cells (Duhen & Campbell, 2014; Paradis et al., 2014; Wong et al., 2013). The interaction of CCR6 and its distinct ligand CCL20 mediated the migration of immune cells into immune reaction sites (Chen et al., 2011; Kallal et al., 2010). Recent evidence showed that CCR6 was also functionally expressed on CD4^+^CD25^+^ regulatory T cells (Tregs) (Rivino et al., 2010). And CCR6^+^ subset of Tregs, a demonstrated memory/effector phenotype, played an important role in various immune diseases (Kitamura, Farber & Kelsall, 2010). Kleinewietfeld et al. (2005) reported that CCR6^+^ Tregs were involved in the pathogenesis of experimental allergic encephalomyelitis (EAE). In tumors, Lamprecht et al. (2008) reported that CCR6^+^ Tregs might favor immune escape of Hodgkin/Reed-Sternberg (HRS) cells. Similarly, our recent work further showed that CCR6^+^ subset of Treg cells were dominantly enriched in tumor mass and closely related to poor prognosis of breast cancer patients (Xu et al., 2010). Notably, the predominant proliferation triggered by DCs was critical for their enrichment and suppressive capacity in tumor mass (Xu et al., 2011). However, the exact regulation mechanism involved in the biological function including proliferation and suppressive capacity of this Tregs subset remains largely unknown; it might be helpful to understanding the contribution of distinct Treg subsets to immunosuppression and ultimately aid the designing of therapy for clinical related disease.

MicroRNAs (miRNAs) are endogenous, non-coding single-stranded RNAs that are approximately 20 nucleotides in length, and have emerged as a key regulator in physiology as well as pathology attributable to its ability to downregulate gene expression through mRNA destabilization/degradation and translation repression by binding onto either 3′ UTR of the target mRNA. Recent studies have shown that different immune cells express distinct miRNA patterns and these miRNA molecules have the ability to modify the expression of target genes and subsequently regulate the function of immune cells (Johanson et al., 2014; Danger et al., 2014; Gigli & Maizon, 2013). For example, miR-21 was highly expressed in CD4^+^ T cells *(Sommers et al., 2013)*, and silencing of miR-21 could alter the proliferation and function of CD4^+^ T cells (Wang et al., 2014). However, whether CCR6^+^ Tregs also expressed specific miRNA patterns and the potential role of these miRNAs in the biological function of these cells remains to be elucidated.

To this end, in the present study, the expression pattern of miRNAs in the CCR6^+^ Tregs was evaluated. Moreover, the mRNA expression profile which might be affected by these miRNAs also was investigated. Our data showed that CCR6^+^ Tregs expressed distinct miRNA signatures which associated with different expression of related genes. These findings might provide novel insight in the role of miRNAs in the function of distinct subset of Tregs.

## Material and Methods

### Animals

Female Balb/c mice 5–6 weeks of age were purchased from the Center of Experimental Animal, Fudan University (Shanghai, China). All animals were housed in the pathogen free mouse colony at our institution and all animal experiments were performed according to the guidelines for the Care and Use of Laboratory Animals (Ministry of Health, PR China, 1998) and all experimental procedures were approved by the Zunyi Medical College Laboratory Animal Care and Use Committee (No. 20130108).

### Flow cytometry

Flow cytometry was performed on a FACSAria (BD Biosciences) with CellQuest Pro software using directly conjugated mAbs against the following human or murine markers: CD4-PerCP, CD25-allophycocyanin, and CCR6-FITC with corresponding isotype-matched controls (either BD Biosciences or eBioscience Systems). Foxp3 staining was conducted using the Murine Regulatory T cell staining kit (eBioscience) and run according to the manufacturer’s protocol.

### miRNA microarray

All sample labeling and GeneChip procession were performed in Kangchen Biotech Corp (Guangzhou, China). One microgram of total RNA was labeled and then hybridized to miRCURY LNA™ microRNA, 7.0 arrays for 16 h at 48 °C. All washing steps were performed by a GeneChip Fluidics Station 450 and GeneChip were scanned with the GeneChip Scanner 3,000 7G. Partek was used to determine ANOVA *p*-values and fold changes for miRNAs. Data is accessible at the NCBI GEO database (accession GSE60041). Species annotations were added and used to filter only those miRNAs found in *Mus musculus*.

### Gene expression microarray

Total RNA was first converted to cDNA, followed by *in vitro* transcription to make cRNA. 5 µg of single stranded cDNA was synthesized; end labeled and hybridized, for 16 h at 45 °C, to Mouse Gene 1.0 ST arrays. All washing steps were performed by a GeneChip Fluidics Station 450 and GeneChip were scanned with the Axon GenePix 4000B microarray scanner. Partek was used to determine ANOVA *p*-values and fold changes for genes.

### Real time PCR

All reagents, primers, and probes were obtained from Applied Biosystems. A U6 endogenous control was used for normalization. Reverse transcriptase reactions and real-time PCR were performed according to the manufacturer’s protocols (Applied Biosystems). RNA concentrations were determined with a NanoDrop instrument (NanoDrop Technologies). One nanogram of RNA per sample was used for the assays. All RT reactions, including no-template controls and RT minus controls, were run in triplicate in GeneAmp PCR 9700 Thermocycler (Applied Biosystems). Gene expression levels were quantified using the ABI Prism 7900HT sequence detection system (Applied Biosystems). Relative expression was calculated using the comparative threshold cycle (Ct) method. The primers used for target genes: murine miR-142a (fwd):5′-TGGCATGAGGATCAGCAGGG-3′, murine miR-142a (rev):5′–GGCAGTCCGCAGCTCTAGG-3′; murine miR-21 (fwd): 5′-GCGTGCTAATGGTGGA-3′, murine miR-21 (rev): 5′-CAGGCGTATCAGTGGG-3′.

### Statistical analyses

Statistical analyses of the data were performed with the aid of analysis programs in SPSS12.0 software. Statistical evaluation was performed using one way analysis of variance (ANOVA) or *t* test using the program PRISM 4.0 (GraphPad Software Inc., San Diego, CA, USA). The *p* values <0.05 were considered significant and are indicated on the figures accompanying this article as follows unless otherwise indicated: ^∗^*p* < 0.05. Unless otherwise indicated, error bars represent SD.

## Results

### MicroRNA expression profiles in CCR6^+^ Tregs

**Figure 1:**
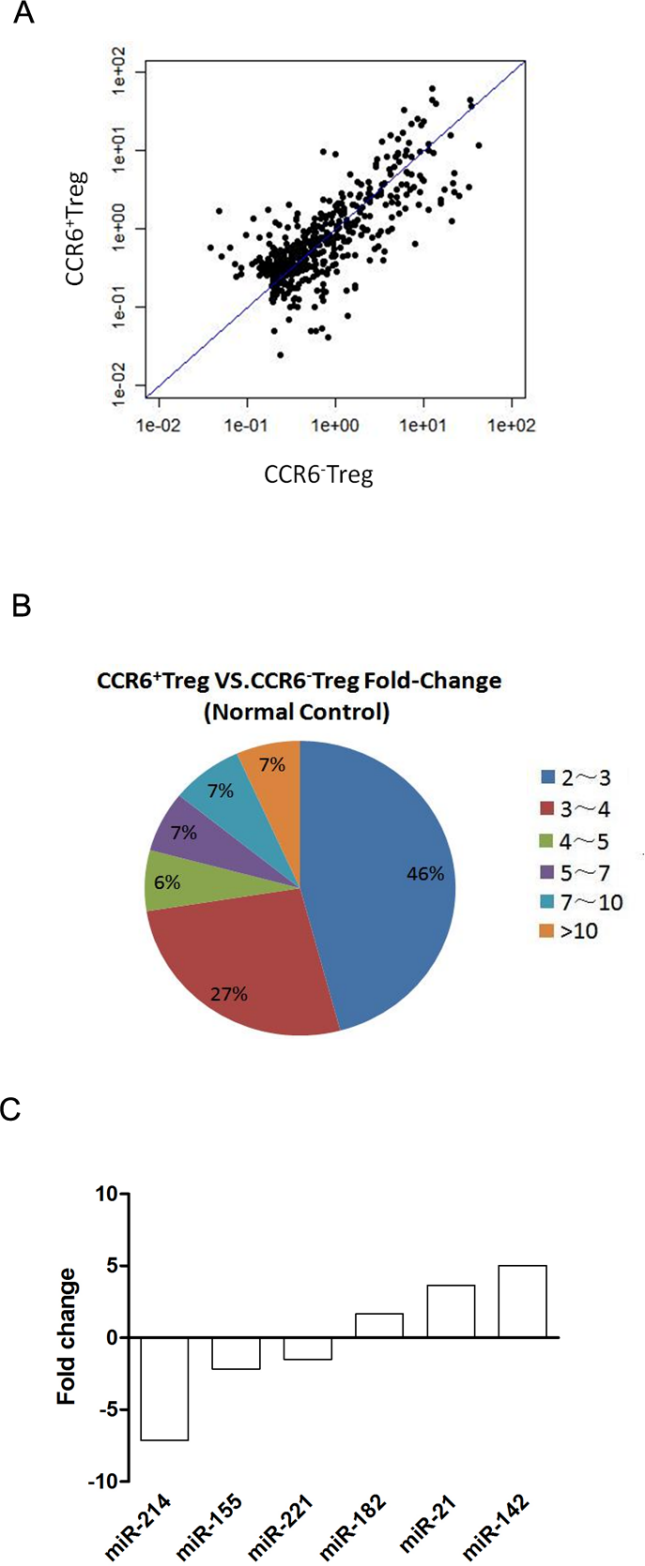
miRNA expression in CCR6^+^ Tregs. CCR6^+^ Tregs and CCR6^-^ Tregs were purified from splenocytes in Balb/c mice. The expression of miRNAs in cells was analyzed by microarray array. (A) A scatterplot of miRNA microarray. (B) A pie graph of miRNA distribution. (C) Predication of the 6 putative miRNAs associated with potential proliferation activity of CCR6^+^ Tregs based on functional similarity of target sets.

Our previous data showed that CCR6^+^ Tregs were dominantly enriched in tumors, which was associated with their potential proliferation activity compared with their CCR6^-^ counterpart (Xu et al., 2010; Xu et al., 2011). In order to characterize the miRNA expression profile that regulates genes involved in potential proliferation activity of CCR6^+^ Tregs, we performed a microarray assay using Affymatrix: GeneChip miRNA 3.0 Array that contains 1,111 mouse probe sequences. Microarray assays showed that miRNA were expressed differentially in CCR6^+^ Tregs. A total of 120 miRNA were significantly altered with the criteria of 2.0 fold change with *p* < 0.05 (Table 1). Out of the 120 altered miRNAs, 58 were upregulated in CCR6^+^ Tregs compared with CCR6^-^ Tregs. As shown in a pie graph of miRNA distribution based on their fold changes in expression (Fig. 1A), the majority of miRNAs altered (88 out of 120) fell into the range of 2.0–4.0 fold up or downregulation. Only eleven miRNAs (five up-regulated and another six down-regulated) displayed over 10 fold changes between two groups (Fig. 1B).

**Table 1:** 120 miRNAs altered in CCR6^+^ Tregs.

miRNA	Fold change	miRNA	Fold change	miRNA	Fold change
mmu-miR-30e-5p	35.12	mmu-miR-344d-3p	2.35	mmu-miR-881-3p	0.37
mmu-miR-27a-3p	14.92	mmu-miR-1983	2.34	mmu-miR-1948-5p	0.37
mmu-miR-5117-3p	13.35	mmu-miR-1947-3p	2.27	mmu-miR-140-5p	0.36
mmu-miR-29b-3p	11.52	mmu-miR-3084-5p	2.25	mmu-miR-3080-5p	0.35
mmu-let-7a-5p	10.21	mmu-miR-467c-3p	2.25	mmu-miR-130b-3p	0.33
mmu-miR-425-5p	8.82	mmu-miR-3084-5p	2.25	mmu-miR-466e-5p	0.32
mmu-miR-29a-3p	8.8	mmu-miR-467c-3p	2.25	mmu-miR-467e-3p	0.32
mmu-miR-181a-5p	8.43	mmu-miR-691	2.24	mmu-miR-668-5p	0.32
mmu-miR-25-3p	5.99	mmu-miR-691	2.24	mmu-miR-24-2-5p	0.31
mmu-miR-19b-3p	5.74	mmu-miR-297c-5p	2.23	mmu-miR-467g	0.3
mmu-miR-142-3p	5.03	mmu-miR-1193-3p	2.19	mmu-let-7g-5p	0.29
mmu-miR-5105	4.74	mmu-miR-767	2.17	mmu-miR-669b-3p	0.29
mmu-miR-744-5p	4.15	mmu-miR-5625-3p	2.14	mmu-let-7d-3p	0.28
mmu-miR-712-5p	3.83	mmu-miR-673-3p	2.13	mmu-miR-3068-5p	0.28
mmu-let-7c-5p	3.73	mmu-miR-207	2.08	mmu-miR-431-5p	0.28
mmu-miR-21a-3p	3.39	mmu-miR-670-5p	2.07	mmu-miR-3473b	0.28
mmu-miR-3474	3.37	mmu-miR-465a-5p	2.05	mmu-miR-30b-5p	0.28
mmu-miR-3096b-5p	3.27	mmu-miR-28a-3p	2.03	mmu-miR-669i	0.27
mmu-miR-3470a	3.16	mmu-miR-1900	2.02	mmu-miR-1843a-3p	0.27
mmu-miR-3097-5p	3.07	mmu-miR-1935	2.01	mmu-miR-32-5p	0.25
mmu-miR-3097-5p	3.07	mmu-miR-5616-3p	0.5	mmu-miR-127-3p	0.24
mmu-miR-3097-5p	3.07	mmu-miR-881-5p	0.5	mmu-miR-29a-5p	0.23
mmu-miR-665-3p	3.05	mmu-miR-30e-3p	0.49	mmu-miR-669c-5p	0.23
mmu-miR-665-3p	3.05	mmu-miR-425-3p	0.49	mmu-miR-329-3p	0.21
mmu-miR-665-3p	3.05	mmu-miR-340-3p	0.47	mmu-miR-30d-5p	0.2
mmu-miR-466j	3.03	mmu-miR-500-3p	0.47	mmu-miR-3084-3p	0.19
mmu-miR-466j	3.03	mmu-miR-467h	0.46	mmu-miR-466d-5p	0.19
mmu-miR-466j	3.03	mmu-miR-669a-3-3p	0.45	mmu-miR-3962	0.17
mmu-miR-491-3p	3.02	mmu-miR-669d-5p	0.44	mmu-miR-3069-5p	0.17
mmu-miR-466f-5p	2.95	mmu-miR-467f	0.44	mmu-miR-669p-3p	0.16
mmu-miR-5099	2.94	mmu-miR-30c-5p	0.44	mmu-miR-3082-5p	0.15
mmu-miR-2137	2.94	mmu-miR-144-3p	0.44	mmu-miR-423-5p	0.14
mmu-miR-26a-5p	2.88	mmu-miR-467e-5p	0.44	mmu-miR-669e-5p	0.12
mmu-miR-26b-5p	2.84	mmu-miR-191-5p	0.43	mmu-miR-374b-5p	0.11
mmu-miR-1971	2.74	mmu-miR-466a/b/c/e/p-3p	0.43	mmu-miR-3096a-3p	0.1
mmu-miR-3473a	2.63	mmu-miR-665-5p	0.42	mmu-miR-466i-5p	0.1
mmu-miR-5129-5p	2.61	mmu-miR-3095-5p	0.41	mmu-miR-1231-3p	0.1
mmu-miR-592-3p	2.53	mmu-miR-466f	0.41	mmu-miR-467b-5p	0.09
mmu-miR-5627-5p	2.5	mmu-miR-511-3p	0.38	mmu-miR-1843b-5p	0.06
mmu-miR-33-5p	2.44	mmu-miR-5616-5p	0.37	mmu-miR-222-3p	0.05

To further investigate which miRNAs was potentially involved in the proliferation activity of CCR6^+^ Tregs, 6 miRNAs among 120 altered miRNAs, which was well documented related to the proliferation activity of T cells, was shown (Fig. 1C). In addition, we further confirmed the expression of miR-142a and miR-21 in these 6 miRNAs by quantitative PCR. Data showed that the expression of miR-142a and miR-21 were also significantly upregulated in CCR6^+^ Tregs compared with those in CCR6^-^ Tregs respectively (Fig. S1, *p* < 0.05), which was consistent with the data in miRNA array.

### Gene expression profile and signaling pathway in CCR6^+^ Tregs

**Figure 2:**
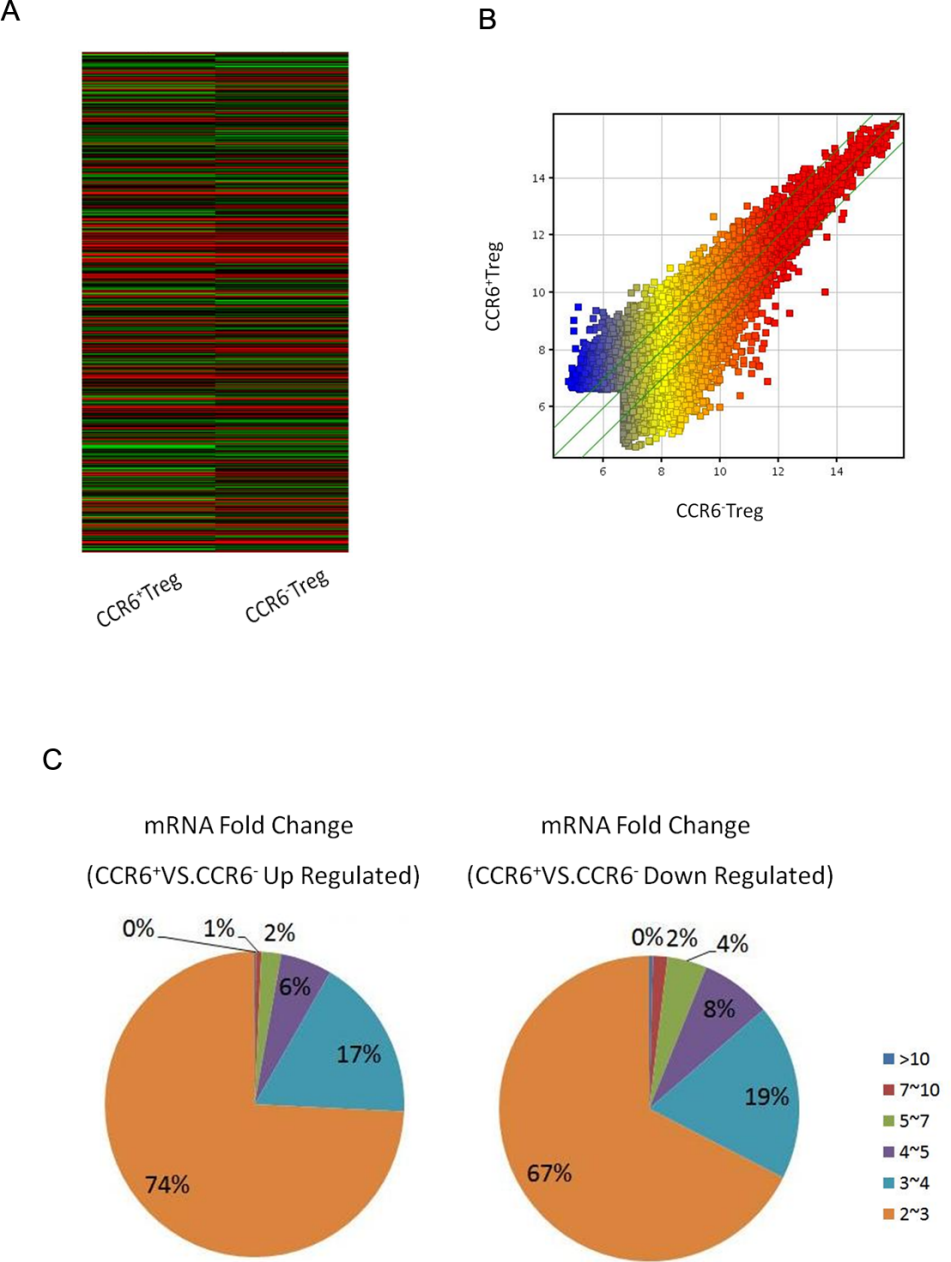
Gene expression in CCR6^+^ Tregs detected by microarray assays. CCR6^+^ Tregs and CCR6^-^ Tregs were purified from splenocytes in Balb/c mice. The global expression of genes in cells was analyzed by microarray array. (A) A heat map of miRNA microarray. (B) The scatterplot for the variation between CCR6^+^ Tregs and CCR6^-^ Treg. (C) The fold change and frequency between CCR6^+^ Tregs and CCR6^-^ Tregs.

To investigate the possible function of these altered expression miRNA molecules in CCR6^+^ Tregs, we detected the global gene expression changes in CCR6^+^ Tregs. CCR6^+^ Tregs and CCR6^-^ Tregs were harvested and subjected to gene expression microarray assay. The altered gene expression profiles in CCR6^+^ Tregs were shown in a heat map (Fig. 2A). Given a three-fold change and *p* < 0.05 (up and down) in differential expression as a cut-off, the number of altered genes was reduced to 1,391; 651 of them were downregulated, and 740 genes were up regulated (Tables 2 and 3).

**Table 2:** Over 3-fold up-regulation genes (651) in CCR6^+^ Tregs.

Target gene	Fold change	Target gene	Fold change	Target gene	Fold change
Kcnh7	21.32	Aurkb	3.94	Fam195b	3.33
Olfr250	16.83	Faim3	3.93	Sept11	3.32
Gm11623	13.12	AU022751	3.93	Chi3l3	3.32
Treml4	12.86	Igh	3.92	Adora3	3.32
Dcn	11.30	Gda	3.92	Tcf7l2	3.32
Gm13766	10.31	Olfr777	3.92	Sdc1	3.32
Rpap1	9.22	Gm4698	3.91	Cecr2	3.32
V1rc16	9.14	Wdr66	3.91	A2ld1	3.32
Dlgap5	9.12	S100a16	3.91	Haao	3.32
N/A	8.35	Cd22	3.91	AW146020	3.32
Cts6	8.32	2610035F20Rik	3.91	Veph1	3.32
Atp6v1b1	8.24	Igh	3.91	N/A	3.32
Dnahc12	8.24	Kiss1	3.91	Dhdds	3.31
Adam29	8.17	Brdt	3.90	H2-Ab1	3.31
AW551984	8.11	Pycrl	3.89	N/A	3.31
Hvcn1	8.04	Gm2987	3.89	Vmn2r38	3.31
D630033O11Rik	7.87	Igh	3.88	2010204K13Rik	3.31
4933405L10Rik	7.59	Npepps	3.88	Cd72	3.31
Igh	7.50	Clip2	3.87	Gzmb	3.30
Fam131b	7.23	Gm3758	3.87	Zfp385b	3.30
Ly6g5b	7.15	Gys1	3.87	Pdgfra	3.30
Klra13	6.71	N/A	3.87	1700057H15Rik	3.30
Neurod6	6.62	Xrcc1	3.87	Txndc17	3.29
Mef2c	6.62	N/A	3.86	Etv5	3.29
P2ry4	6.43	Zfp553	3.85	Fcer1g	3.29
Neil3	6.39	Nol9	3.85	Gm14920	3.29
Trappc2	6.39	Tsen54	3.85	Olfm4	3.29
Tmem109	6.38	Ints7	3.85	N/A	3.29
Clec4n	6.38	Tcfeb	3.85	Eral1	3.28
Vmn2r102	6.36	P2ry1	3.84	2310030N02Rik	3.28
N/A	6.36	Hist1h2bg	3.84	Gm13403	3.28
Gm10649	6.32	Mxd1	3.84	Idh3b	3.28
Cage1	6.31	Cadps2	3.83	Chd5	3.28
Gtf2ird2	6.28	N/A	3.83	Tssk2	3.28
N/A	6.27	2310061I04Rik	3.83	Cbwd1	3.28
Eya1	6.22	Fcer2a	3.82	Robo1	3.27
Mpo	6.09	Klhl13	3.82	Whsc1	3.27
Gpr152	6.07	Pah	3.82	Bmp1	3.27
AI324046	6.04	Zdhhc3	3.81	Pygl	3.27
Ccdc82	5.99	Lcn2	3.81	Pvrl4	3.27
4933411K16Rik	5.98	Zbtb34	3.81	Cd180	3.27
Pigt	5.96	Sirpb1	3.81	Tpsg1	3.26
Havcr2	5.94	Adam1a	3.79	Gprc5a	3.26
4933402D24Rik	5.92	Ace2	3.79	Gm13375	3.26
Myom1	5.92	C86187	3.79	1810034E14Rik	3.26
Kif2c	5.72	March4	3.79	Il1b	3.26
Olfr514	5.65	Pigq	3.79	C330016O10Rik	3.26
Gm7306	5.62	Lingo1	3.78	Ank2	3.25
Dnajc28	5.59	Nuak2	3.78	Ins2	3.25
4930578G10Rik	5.58	V1rd2	3.77	Hrh4	3.25
4930517G24Rik	5.57	Igh	3.77	Trp53rk	3.25
Gm12260	5.57	Cdc20	3.77	Grik1	3.24
N/A	5.53	Adam9	3.76	Asgr1	3.24
Gm2847	5.53	Gm13152	3.76	Lrrc59	3.24
Gp49a	5.50	Ccnf	3.76	N/A	3.24
Fcamr	5.49	Csgalnact2	3.76	2810408A11Rik	3.24
Klhdc7b	5.48	Vps53	3.75	Gcet2	3.24
Cacna1f	5.46	Uggt2	3.74	Lrrk2	3.24
4930467D21Rik	5.42	Rbm8a	3.73	Pira11	3.24
Masp1	5.34	Igk	3.73	Tusc1	3.24
N/A	5.32	Pcbp1	3.73	Usp35	3.24
Stk33	5.32	Klk15	3.73	Panx3	3.24
Xirp1	5.31	Smox	3.73	Vti1a	3.23
Prune	5.30	Gm5393	3.73	Nudt16l1	3.23
Brpf1	5.27	Txnl4b	3.72	Tnk1	3.23
Zdbf2	5.26	9130017N09Rik	3.72	Ighv14-2	3.23
4930432E11Rik	5.24	Rims1	3.72	Hspb11	3.23
Arhgap24	5.24	Spire1	3.71	Blk	3.23
N/A	5.22	N/A	3.71	Zdhhc4	3.23
Il15	5.20	Psmg4	3.70	Phka1	3.22
Plin1	5.18	Mrps36	3.70	Micalcl	3.22
Spink10	5.18	Pstk	3.70	Gm13089	3.22
Snca	5.15	Trmt2a	3.70	RP23-480B19.10	3.22
Styxl1	5.14	Nsg2	3.70	Rwdd3	3.22
Ranbp17	5.14	Anxa1	3.70	1110037F02Rik	3.22
Mcam	5.09	Lpcat2	3.69	Krtap13	3.21
Vmn2r121	5.09	Asb4	3.69	Cd22	3.21
Chi3l4	5.08	Sprr2a3	3.69	Hist1h2ab	3.21
Ltb4r2	5.02	Rps6kb1	3.69	2700008G24Rik	3.21
Ppp1r3d	5.02	Zfp282	3.68	N/A	3.21
Gm2705	5.00	Wdfy4	3.68	Chst14	3.21
Etl4	4.98	Gm2448	3.68	A2bp1	3.20
Fam108b	4.93	Lta4h	3.67	Gm2739	3.20
Adamts8	4.92	1600020E01Rik	3.67	Lman1	3.20
Akr1c13	4.91	Psg29	3.66	Timp1	3.20
Gm11543	4.89	Sik3	3.66	Rad54b	3.20
Il17c	4.89	4933421E11Rik	3.65	1700012C08Rik	3.20
Ccdc30	4.89	Ltf	3.65	LOC668727	3.20
Tmed9	4.88	Lpp	3.65	Sytl3	3.20
Fam46a	4.87	H2-Aa	3.65	Zfp710	3.19
N/A	4.87	Gm2586	3.64	Pex11b	3.19
Clic5	4.86	Lphn3	3.64	Ncf1	3.19
Gm5153	4.85	A530023O14Rik	3.64	Sh3pxd2a	3.19
Fzd1	4.84	Msh5	3.64	Ush2a	3.19
Hemt1	4.82	Gm11981	3.64	Trim29	3.19
Anxa1	4.79	Crem	3.64	Pecam1	3.18
Retnlg	4.78	Lmo2	3.63	Mtus1	3.18
Gm7219	4.77	Gm4846	3.63	Fam55b	3.17
Tmem63b	4.77	Apoo	3.63	Gm2461	3.17
Clec4d	4.75	Btbd7	3.63	Golim4	3.17
4933416M06Rik	4.73	Med8	3.62	Acp1	3.17
Zyx	4.73	Mgl1	3.62	Gm2695	3.17
Klk1b4	4.72	Med31	3.62	Kdelc2	3.17
Defb30	4.71	Abca16	3.61	Myo1c	3.17
Insc	4.65	Hes6	3.61	Gprc5b	3.16
Hs3st2	4.65	Igh	3.61	Rcn3	3.16
Ubap1	4.62	Cdkl5	3.60	Rassf4	3.16
Gpr56	4.61	Oxgr1	3.60	Adrb2	3.16
Igh-VJ558	4.61	F5	3.60	Cd36	3.16
Igh	4.61	Psmd13	3.59	Slc34a3	3.15
Cpne2	4.61	Clock	3.59	Acot4	3.15
2610028H24Rik	4.60	Stab1	3.58	Ccdc157	3.15
Rasl10a	4.58	Coasy	3.58	Igl-V1	3.15
Mrpl33	4.58	Fcrla	3.57	4930534B04Rik	3.15
Fn3k	4.58	Cybb	3.56	Gm6127	3.15
9430025M13Rik	4.57	D2hgdh	3.56	3110056O03Rik	3.15
Gm13083	4.55	Igh	3.56	Kcnb2	3.15
Klra6	4.54	Adamtsl1	3.56	Atp8b4	3.15
4933412E24Rik	4.53	BC005705	3.56	Gm10883	3.15
Zfp707	4.52	Loxl4	3.56	Bcr	3.14
Rapgefl1	4.52	Ncapd2	3.55	Mtus1	3.14
Scyl2	4.50	Hdc	3.55	Sgsm3	3.14
Rab7l1	4.49	Gem	3.55	Tdp1	3.14
Scfd1	4.49	N/A	3.55	Tcf15	3.14
N/A	4.48	Sepx1	3.55	Lmbr1	3.14
Gm4395	4.48	Tubgcp5	3.54	Ermap	3.14
Odf4	4.46	Cpne2	3.54	2210009G21Rik	3.14
Nfam1	4.46	Rarres1	3.54	N/A	3.13
Topbp1	4.46	Ebf3	3.54	Dhx35	3.13
Grhl1	4.46	Csf1r	3.54	Ell3	3.13
Guf1	4.45	N/A	3.54	4930406D18Rik	3.13
Trpm3	4.44	Igh	3.54	Ubd	3.13
Ciita	4.43	N/A	3.54	Gm6425	3.13
Hist1h2ak	4.42	Mfsd3	3.53	Hist1h3e	3.13
Igh	4.42	Homer2	3.53	Slc22a17	3.13
Fcgr2b	4.42	Zbtb16	3.53	Serpinb1c	3.12
Wac	4.42	Ifltd1	3.52	Sln	3.12
Msmb	4.41	Gm10693	3.52	Gm10766	3.12
Plac8	4.41	Ptgs1	3.52	Adipor1	3.12
Nr5a1	4.38	Sh2d3c	3.51	Gm684	3.11
Gm13446	4.37	V1rc29	3.51	Il1f9	3.11
Vmn2r73	4.37	Lrp1	3.51	Kcnj16	3.10
Pfkfb4	4.37	Nova1	3.51	Car1	3.10
Phyhipl	4.36	N/A	3.51	Psme4	3.10
Gpatch4	4.36	4930578N18Rik	3.49	Siglec5	3.10
Cenph	4.36	A030001D20Rik	3.49	N/A	3.10
Gm13154	4.35	Hsf4	3.49	Igk-C	3.10
Tm2d1	4.35	Trem3	3.49	N/A	3.10
Ptplad2	4.35	Arhgap24	3.48	Igh	3.10
Gm13597	4.34	Lins2	3.48	2310002J15Rik	3.09
Nkd1	4.34	Igh	3.48	G630018N14Rik	3.09
Phox2b	4.33	Prnd	3.48	Rbx1	3.09
Cyp2j7	4.33	4930529M08Rik	3.47	Gm8787	3.08
Pstpip2	4.31	3110009E18Rik	3.47	N/A	3.08
Fam81b	4.29	Hist1h2bb	3.46	Gm7170	3.08
Pira3	4.29	Ncapg	3.46	Cd19	3.08
Gpr112	4.28	E030019B13Rik	3.46	Wfdc1	3.08
5031414D18Rik	4.27	Gm3528	3.46	Casp12	3.08
Trpm3	4.27	Gm15498	3.46	6330416G13Rik	3.07
Slco4c1	4.27	Cryz	3.46	Il6ra	3.07
Zfp354b	4.25	Stard4	3.46	Scd1	3.07
Camp	4.24	Bfsp2	3.45	H2afy	3.07
Ric3	4.24	Rpap1	3.45	Lmbrd1	3.07
Tsfm	4.23	Vsig1	3.44	Pira1	3.07
Abcc3	4.22	Olfr1431	3.44	Gm5468	3.07
BC035044	4.22	Abcb4	3.44	Pgap1	3.07
C230096C10Rik	4.22	Vwc2	3.44	Prom2	3.07
Nkg7	4.20	Rpap1	3.44	Nubp1	3.07
Gm15623	4.20	5830477G23Rik	3.43	C1qb	3.07
Casc1	4.20	Gypa	3.43	Tcf7l2	3.06
Lsm1	4.19	Slc25a42	3.43	Ebf1	3.06
Anxa6	4.19	Arhgap26	3.43	Itgb6	3.06
D130009I18Rik	4.17	Ccl6	3.42	Terf2	3.06
Il1b	4.17	Cbfa2t3	3.42	Prosc	3.06
Pcdh17	4.16	Snx29	3.42	N/A	3.06
Clec4d	4.16	Ube2w	3.42	Il9r	3.05
Alk	4.16	Slc1a1	3.41	Gm14206	3.05
Cd79b	4.15	Olfr399	3.41	Fignl1	3.05
Zc3h7b	4.15	D930016D06Rik	3.41	Dhrs3	3.05
Mc4r	4.15	Hs2st1	3.41	Ikbkg	3.05
Sept8	4.13	Pou3f3	3.41	Map3k7ip1	3.05
Gp49a	4.13	Ccdc46	3.41	Lcat	3.05
N/A	4.12	Olfr1434	3.41	Itsn1	3.05
Smarcd1	4.12	Pcdh15	3.40	Creld1	3.05
2700050L05Rik	4.11	N/A	3.40	Gm9121	3.04
Fmnl2	4.11	Ctbp2	3.40	Klrb1c	3.04
Gm11686	4.11	Pla2g7	3.40	Gpr116	3.04
Ube1y1	4.10	Clk2	3.40	Igh-6	3.04
1600012P17Rik	4.10	Gen1	3.40	Igk-C	3.04
Irf5	4.09	Stoml1	3.39	Cstf1	3.04
Caskin1	4.08	Prpf19	3.39	Cel	3.04
Cd300lf	4.08	Acer2	3.39	Slc30a1	3.04
Oosp1	4.07	Rhox2c	3.39	N/A	3.04
Xlr3a	4.07	Snn	3.38	Gm10193	3.03
Nol4	4.07	V1rb8	3.38	Gm9880	3.03
Map2k7	4.06	Sema4a	3.38	N/A	3.03
Gm5577	4.05	Tmeff1	3.38	Gm2436	3.03
Trmt12	4.04	Olfr395	3.38	Prr14	3.03
Sec14l1	4.04	LOC677563	3.38	Spsb1	3.03
D930015E06Rik	4.03	Rfc2	3.37	Hbb-b2	3.03
Slpi	4.03	A430075N02	3.37	Acrv1	3.02
Gga1	4.03	Pvrl2	3.36	Shmt1	3.02
Tex101	4.03	Snx8	3.36	Bcl11a	3.02
Itsn1	4.02	Adamts1	3.36	N/A	3.02
Gm3323	4.02	Pnmt	3.36	Ly6g	3.02
Gm2954	4.01	Poll	3.36	Cd74	3.02
Slc35e4	4.01	Serpina1f	3.35	Fchsd2	3.02
C1qa	4.00	Pla2g12a	3.35	Pik3cg	3.02
Retnlg	4.00	Kel	3.35	3300005D01Rik	3.01
Cul2	3.99	Cks2	3.35	Prc1	3.01
Plekhm3	3.99	Axl	3.35	Hyou1	3.01
Cyth2	3.98	2010110P09Rik	3.34	Gnb2	3.01
Scfd2	3.98	Spink12	3.34	Pla2g15	3.01
Gns	3.98	4933400N17Rik	3.33	2010308F09Rik	3.01
Yif1a	3.96	Cd300lf	3.33	Gm10270	3.00
N/A	3.95	Hist1h4f	3.33	Pak7	3.00
N/A	3.95	Zfp800	3.33	C730027P07Rik	3.00

**Table 3:** Over 3-fold down-regulation genes (740) in CCR6^+^ Tregs.

Target gene	Fold change	Target gene	Fold change	Target gene	Fold change
Il2ra	25.65	Atxn7l1	4.27	Trim37	3.41
N/A	18.99	Gm5282	4.25	Ksr2	3.41
Gm9119	15.47	St3gal3	4.25	ENSMUSG00000079376	3.41
Il2ra	15.05	4930417O13Rik	4.24	Ptpn5	3.40
N/A	14.55	Trerf1	4.24	9230117E06Rik	3.40
Ctla4	14.24	Klk6	4.23	N/A	3.40
Gal3st1	12.37	2610042L04Rik	4.22	N/A	3.40
Gm3453	12.21	Cyp4f41-ps	4.22	Plekha1	3.39
Gal	12.20	Clcn1	4.21	Trav3n-3	3.39
ENSMUSG00000072735	11.93	Abcb7	4.20	Lrsam1	3.39
Foxp3	11.69	Bcs1l	4.20	Olfr109	3.39
Cyb5r2	11.65	Stk19	4.18	Rsbn1	3.39
Phkg1	10.53	Sectm1a	4.18	Odf1	3.39
Ikzf2	10.44	Fmr1nb	4.17	Mc2r	3.38
Evc2	10.17	Pnkd	4.17	Ifna6	3.38
Il17rc	10.00	N/A	4.17	Gm7223	3.38
Plekhg5	9.93	Gpr110	4.17	Cntn4	3.38
ENSMUSG00000072735	9.66	Inpp4b	4.17	N/A	3.38
Acer2	9.56	Gatsl3	4.17	Gm10228	3.38
Neb	9.55	Dapk1	4.16	Gm5169	3.37
Gpr45	9.49	Gm3455	4.15	R3hcc1	3.37
D15Wsu169e	9.47	Gm14717	4.14	Slc38a1	3.37
Brunol5	9.44	1700001E04Rik	4.14	Inpp4b	3.37
Pxdn	9.44	Pde4a	4.13	Nphp3	3.37
Gpr83	9.43	Slc35f2	4.13	Csnk1g1	3.36
ENSMUSG00000072735	9.43	Adam6b	4.13	Jazf1	3.36
Gm3727	9.36	Penk	4.13	Arhgdig	3.36
Gm3727	9.25	2510048L02Rik	4.13	Etaa1	3.36
N/A	9.24	Casp3	4.12	Cul2	3.36
Gm11744	9.05	Dcaf17	4.12	Gm10837	3.36
Gm3339	8.66	Gm3182	4.12	Ppp2r3a	3.36
Dpy19l2	8.60	1500015O10Rik	4.11	Gm1574	3.35
Caskin2	8.31	Acsl4	4.11	Tspan12	3.35
Ikzf2	8.22	Ddx43	4.10	Magi3	3.35
Tubgcp5	8.17	AI987944	4.09	1110059M19Rik	3.35
Gm2974	8.16	Plin1	4.09	Cpsf4l	3.34
C230088H06Rik	8.08	Tox	4.09	Parp4	3.34
Fbxw27	8.05	Gm10338	4.07	Galr3	3.34
Gm14005	8.04	Zscan12	4.06	Adam33	3.34
Gm8362	7.95	Fam71e1	4.06	Frs3	3.33
Gm8297	7.93	Neb	4.06	Ptgdr	3.33
Pla2g2d	7.93	100039441	4.05	BE691133	3.33
Slc22a12	7.91	BC106179	4.05	Brp44l	3.33
N/A	7.87	N/A	4.05	Gm11468	3.33
Cadm3	7.81	Stab1	4.04	Dctn4	3.33
Cyhr1	7.58	Tnfsf13b	4.04	E330021D16Rik	3.33
B630019K06Rik	7.58	Mdfi	4.03	Gm3764	3.32
Inpp4b	7.50	A930002C04Rik	4.03	Cd300lg	3.32
Ctla4	7.49	Slc23a3	4.03	Atg2a	3.32
Cyp2u1	7.47	Col6a3	4.02	Ankrd9	3.32
Gm3182	7.44	Ghrh	4.01	Gm7225	3.32
Tgfb2	7.43	A930017M01Rik	4.01	Pnpla7	3.32
Vwce	7.41	Itih5l	4.01	Cd96	3.31
LOC100036568	7.32	Aurkc	4.00	4833422F24Rik	3.31
1700029I01Rik	7.31	Itga6	4.00	Thnsl2	3.31
Olfr701	7.29	Mfrp	3.99	Pdcd11	3.31
Rfc3	7.29	1700042G15Rik	3.99	Robo4	3.31
Gm10014	7.22	Mageh1	3.98	Aven	3.31
N/A	7.20	Ptpn13	3.98	1700026L06Rik	3.31
LOC100038847	7.16	Olfr227	3.98	Lrig2	3.31
544988	7.09	1700028M03Rik	3.98	Ehbp1	3.31
Gm4489	7.07	Gpatch4	3.98	Kctd9	3.30
LOC100038847	6.95	Pxmp2	3.97	Zbtb37	3.30
Nlrx1	6.94	Mllt3	3.97	Lrrc34	3.30
N/A	6.92	Gm10250	3.97	Zfp30	3.30
Gm3642	6.92	Cux1	3.96	Ano2	3.29
Tgm1	6.90	Csmd1	3.96	N/A	3.29
Dmd	6.88	Ptger3	3.96	Tmem134	3.29
Foxp3	6.85	Gm3990	3.95	Sh2d6	3.29
ENSMUSG00000072735	6.82	2010005J08Rik	3.94	Olfr78	3.29
Gria1	6.82	Olfr623	3.94	Mapk8	3.29
Arhgef15	6.81	ENSMUSG00000072735	3.94	Upp1	3.29
Gm2888	6.79	March7	3.94	Gm2046	3.29
Fdft1	6.73	N/A	3.94	Tex21	3.28
Gm3642	6.72	Slc9a3	3.93	Tnfrsf4	3.28
Nck2	6.70	Rbm9	3.93	Nol11	3.28
Adamtsl4	6.64	Dtwd1	3.93	1700092C10Rik	3.28
Zfp142	6.60	C77370	3.93	Gm3916	3.28
Gm3269	6.59	N/A	3.92	Dmxl2	3.28
Gm3411	6.56	Fbxw13	3.92	ENSMUSG00000072735	3.28
544988	6.53	Amz2	3.92	ENSMUSG00000079376	3.27
9630058J23Rik	6.53	Nsl1	3.92	4930587E11Rik	3.27
2010109N18Rik	6.51	Plxna3	3.92	Plcl1	3.27
N/A	6.51	Ppme1	3.90	Srgap3	3.27
Brap	6.51	Gcgr	3.90	Prss39	3.27
Tmem210	6.47	Sgcd	3.90	Dapk3	3.26
4930486G11Rik	6.46	N/A	3.89	Fbxw24	3.26
Vmn2r46	6.46	ENSMUSG00000068790	3.89	Gm3626	3.26
1110017D15Rik	6.46	Olfr658	3.88	Mtap4	3.26
N/A	6.43	Fbxo15	3.88	Gm3253	3.25
N/A	6.43	Mrgprb4	3.88	Cypt6	3.25
N/A	6.38	Ncoa7	3.87	Aatf	3.25
Gm3518	6.36	Grin1	3.87	Il2rb	3.25
C430002E04Rik	6.26	4933400A11Rik	3.87	Fam160a1	3.24
Gm13620	6.23	Vmn2r72	3.87	Ece1	3.24
Gm3685	6.23	Pfkp	3.87	Nkx2-6	3.24
Zscan10	6.22	Igl	3.86	Pik3r2	3.24
Gm10340	6.22	4930445K14Rik	3.86	Slc25a21	3.24
Gm3159	6.21	Krtap5-4	3.85	Ptgfrn	3.24
5830403L16Rik	6.16	Gm3424	3.85	N/A	3.24
Gm3127	6.15	Cd247	3.85	Tbcel	3.23
B930046C15Rik	6.13	Samsn1	3.85	Sgip1	3.23
Syne2	6.10	Uty	3.84	1700023L04Rik	3.23
Gm3029	6.10	Galk1	3.84	Catsper3	3.23
1600002D24Rik	6.06	1700029G01Rik	3.84	Dgka	3.23
Gm2224	6.04	Agrn	3.83	4930433N12Rik	3.23
Gm4801	6.00	Lrig2	3.83	Rdh16	3.22
N/A	5.97	Slc25a27	3.83	BC011248	3.22
Pitpnc1	5.94	Gjb4	3.83	Dlgap1	3.22
Gm3476	5.94	Rgs16	3.83	Olfr1283	3.22
6430562O15Rik	5.92	Cntn1	3.82	Osbpl3	3.22
Vmn2r66	5.92	Fndc7	3.82	Foxi2	3.21
Gm3029	5.91	Itk	3.82	Fam186a	3.20
Gm3115	5.89	N/A	3.82	Gm8356	3.20
Ndp	5.84	H1fx	3.81	Timp4	3.20
Zfp329	5.83	Pik3c2a	3.80	Tbc1d8	3.20
Gpr64	5.82	Ctsj	3.80	Srd5a1	3.20
Nav2	5.81	Emid1	3.80	Olfr242	3.20
Aven	5.80	Serpina1e	3.79	Sel1l	3.19
D030054H15Rik	5.79	Gm1330	3.79	Mbnl2	3.19
Grik5	5.76	Tbc1d4	3.79	Plac9	3.19
Sgsm3	5.71	Hs1bp3	3.79	N/A	3.19
Ovol2	5.70	Olfr961	3.79	Slc12a1	3.19
Mc1r	5.65	Pask	3.78	Zfp169	3.19
Gm10371	5.65	BC060267	3.78	Dok7	3.18
N/A	5.62	Kpna1	3.78	Gm2275	3.18
Luzp2	5.60	Arg1	3.77	Gm2643	3.18
Pthlh	5.59	Cts8	3.77	Dpep2	3.18
1700021F07Rik	5.58	Suclg1	3.77	Pard6b	3.18
Ccbe1	5.56	1700001E04Rik	3.77	Cyp17a1	3.17
Cul7	5.55	Synpo2	3.77	9330111N05Rik	3.17
Cpped1	5.55	6030458C11Rik	3.77	Ccdc33	3.17
Fmnl3	5.54	1190002H23Rik	3.77	Tub	3.17
D6Wsu163e	5.53	Rpusd3	3.76	Rpl7l1	3.17
Serpinb9d	5.49	Gm6710	3.76	AW495222	3.17
E030025P04Rik	5.49	Ikbkap	3.76	Ipcef1	3.17
Skap1	5.49	N/A	3.76	Tle2	3.17
D0H4S114	5.47	4921523L03Rik	3.75	B3galnt2	3.17
Piwil2	5.45	Speer4f	3.75	Ndel1	3.16
4930524L23Rik	5.45	Gm3047	3.75	Atp6v1c2	3.16
Ykt6	5.43	Synpr	3.74	Hnrpll	3.16
Slc24a3	5.40	N/A	3.73	Prrg1	3.16
N/A	5.39	9030624G23Rik	3.72	Cyp2j13	3.16
Gm6337	5.38	Trp53inp2	3.72	Espn	3.16
Gm3149	5.34	9130401M01Rik	3.72	Mup1	3.15
Tnfrsf9	5.29	Myst4	3.72	Ptprr	3.15
Ttn	5.28	Gm12836	3.72	Snx11	3.15
Gpr52	5.27	2810039B14Rik	3.71	Chchd8	3.15
Cntfr	5.26	Fastk	3.71	Dnm1	3.15
ENSMUSG00000079376	5.25	Inpp4b	3.71	Tbc1d25	3.15
LOC100038847	5.22	N/A	3.70	Olfr1120	3.14
Mybpc2	5.22	Prlh	3.70	Gm3981	3.14
Cdon	5.16	Mcm8	3.70	Morc2a	3.14
Slitrk6	5.16	Gm15340	3.70	Ttll7	3.14
Dom3z	5.14	Gm4926	3.69	Irf6	3.14
Gm3149	5.14	Ebpl	3.69	A830039H05Rik	3.13
Gm16521	5.14	N/A	3.69	1700024B18Rik	3.13
Smarcal1	5.14	4930417O13Rik	3.68	Trap1a	3.13
C230099D08Rik	5.13	Pcdh15	3.68	Vmn2r10	3.13
Olfr1252	5.11	Ctla4	3.68	Nrn1	3.13
4930599N23Rik	5.11	4933432I09Rik	3.68	Mapkapk3	3.13
Gm3642	5.10	Hsd17b2	3.68	3110082J24Rik	3.13
Gm5634	5.09	Fbp1	3.67	Ccdc65	3.13
Cngb1	5.08	Gm5795	3.66	Spag6	3.13
Pax3	5.07	Gm8159	3.66	AI428936	3.12
ENSMUSG00000068790	5.07	Atf7	3.66	Tiam1	3.12
4632404H12Rik	5.05	Kdm4a	3.66	Cenpk	3.12
Vill	5.03	Ocrl	3.66	Rapsn	3.12
Gm8050	5.02	Sgol1	3.65	Tm2d3	3.11
Anks4b	5.00	Prox2	3.65	Tiam1	3.11
St3gal6	4.96	Rnf26	3.65	Tle2	3.11
1700034I23Rik	4.96	N/A	3.64	Wbp11	3.11
Gm3172	4.95	Bub1	3.64	Olfr1128	3.11
Spata18	4.93	Trim63	3.63	Art1	3.11
Plcg1	4.92	Slc6a9	3.62	Grin3a	3.11
Has2as	4.91	Dst	3.61	1700110K17Rik	3.10
Ntn4	4.90	AI428936	3.61	Bcat1	3.10
Skap1	4.88	Marveld2	3.60	Iigp1	3.10
Cyp2c50	4.88	Esrrb	3.60	Pla2g4e	3.10
Cope	4.87	Gm4699	3.60	Rpusd1	3.10
N/A	4.86	Ttyh1	3.59	Olfr638	3.10
Gm3642	4.86	Rgs16	3.59	Agbl2	3.10
Tnfrsf18	4.85	8030463A06Rik	3.59	4921509O09Rik	3.09
N/A	4.82	4930578E11Rik	3.58	Olfr389	3.09
Snhg11	4.81	Cacnb2	3.57	Pcsk4	3.09
Gm6121	4.81	Setd3	3.57	Pou2f1	3.09
Ncoa7	4.80	N/A	3.57	Brcc3	3.09
1700025M24Rik	4.75	Gm2957	3.56	Gm3034	3.09
S100a7a	4.75	Magea3	3.56	Gm8362	3.09
Olfr140	4.74	Syngr3	3.56	D030028A08Rik	3.09
Crem	4.73	Gm3127	3.55	Fam118b	3.08
Gab3	4.72	ENSMUSG00000068790	3.55	Ccdc126	3.08
Ift80	4.71	Tmem176a	3.55	Fbxw4	3.08
Secisbp2	4.69	1700081N11Rik	3.55	Cish	3.08
1110019B22Rik	4.67	N/A	3.55	N/A	3.08
N/A	4.66	9530002K18Rik	3.54	A630023P12Rik	3.08
Gm7750	4.64	1700008F21Rik	3.54	Alox12b	3.07
N/A	4.61	Grhl3	3.54	Hsd3b4	3.07
N/A	4.61	Smc2	3.54	Caskin1	3.07
Gm8026	4.61	Fam46d	3.54	Ank3	3.07
4933407C03Rik	4.61	Mypop	3.54	Helz	3.06
Tmub2	4.59	Spats2	3.53	Taar7b	3.06
Tnfrsf25	4.59	Mpa2l	3.53	Gm3602	3.06
Gm3269	4.57	Nosip	3.53	Gm10094	3.06
Gm8297	4.57	Iigp1	3.53	Ptpn9	3.06
9130230L23Rik	4.56	Wdr52	3.51	1700085B03Rik	3.06
4831440E17Rik	4.55	4833442J19Rik	3.51	Gm7696	3.06
N/A	4.54	Tiam1	3.51	2610002I17Rik	3.06
Maf	4.54	Snapc4	3.51	Cav3	3.06
Gm7894	4.54	Dgat2	3.51	Slc4a8	3.06
4932431H17Rik	4.53	Saps2	3.50	Cacna2d1	3.06
E030046B03Rik	4.53	Tasp1	3.50	St3gal4	3.05
Gm3264	4.51	9930013L23Rik	3.50	Gm5134	3.05
Odz3	4.51	Sectm1b	3.49	Plod2	3.05
Olfr725	4.50	LOC432958	3.49	Gm2282	3.05
Frmd6	4.49	Grik2	3.49	Rpl26	3.05
Reck	4.47	B230216N24Rik	3.49	Ly6g6c	3.05
Cars2	4.47	Pla1a	3.49	Gm3453	3.05
Themis	4.46	Bex1	3.48	Suox	3.05
Msh2	4.46	N/A	3.48	Emilin3	3.05
Olfr1356	4.45	Slc35d1	3.48	4931422A03Rik	3.05
E030010N08Rik	4.44	N/A	3.47	Airn	3.05
Ninj2	4.44	Zfp444	3.47	Gm8301	3.04
Dennd2c	4.44	Kcnab3	3.47	Prss23	3.04
LOC100038847	4.43	Gm9893	3.47	Exoc3l	3.04
Ppp2r3a	4.42	Afm	3.46	Gm3556	3.04
Rsad1	4.42	Tecpr1	3.46	Car12	3.04
Nicn1	4.41	Gm7980	3.46	N/A	3.04
N/A	4.40	V1rc26	3.46	Ipcef1	3.03
Osbpl3	4.38	Pyroxd2	3.46	Gm6160	3.03
Duxbl	4.38	Myo1b	3.45	Stk30	3.03
Olfr1019	4.38	Gemin5	3.45	Txk	3.03
Ripk4	4.37	Dzip1	3.45	Klra4	3.03
Ermp1	4.37	Pabpc3	3.45	Icos	3.03
Sfmbt2	4.33	Olfr781	3.45	Ciapin1	3.02
Gpt2	4.33	Agrn	3.44	Frmd4b	3.02
Myct1	4.32	Fam98c	3.44	Gm3278	3.02
E330026B02Rik	4.31	Fam65a	3.44	Scrn3	3.02
Zbtb16	4.31	Plekhg1	3.44	0610031O16Rik	3.02
N/A	4.29	Pbld	3.44	Brwd2	3.02
2010005H15Rik	4.29	Epb4.1l1	3.43	Numbl	3.02
Rragd	4.28	Zap70	3.43	Raph1	3.02
Ephb3	4.28	Kcnk13	3.43	N/A	3.01
Treh	4.28	Mrgprh	3.43	N/A	3.01
Krt72	4.28	Gm8519	3.42	Klrg1	3.01
Snx16	4.28	Cntfr	3.42	Srd5a1	3.01
Tox	4.28	N/A	3.41		
Il2ra	25.65	Atxn7l1	4.27	Trim37	3.41
N/A	18.99	Gm5282	4.25	Ksr2	3.41
Gm9119	15.47	St3gal3	4.25	ENSMUSG00000079376	3.41
Il2ra	15.05	4930417O13Rik	4.24	Ptpn5	3.40
N/A	14.55	Trerf1	4.24	9230117E06Rik	3.40
Ctla4	14.24	Klk6	4.23	N/A	3.40
Gal3st1	12.37	2610042L04Rik	4.22	N/A	3.40
Gm3453	12.21	Cyp4f41-ps	4.22	Plekha1	3.39
Gal	12.20	Clcn1	4.21	Trav3n-3	3.39
ENSMUSG00000072735	11.93	Abcb7	4.20	Lrsam1	3.39
Foxp3	11.69	Bcs1l	4.20	Olfr109	3.39
Cyb5r2	11.65	Stk19	4.18	Rsbn1	3.39
Phkg1	10.53	Sectm1a	4.18	Odf1	3.39
Ikzf2	10.44	Fmr1nb	4.17	Mc2r	3.38
Evc2	10.17	Pnkd	4.17	Ifna6	3.38
Il17rc	10.00	N/A	4.17	Gm7223	3.38
Plekhg5	9.93	Gpr110	4.17	Cntn4	3.38
ENSMUSG00000072735	9.66	Inpp4b	4.17	N/A	3.38
Acer2	9.56	Gatsl3	4.17	Gm10228	3.38
Neb	9.55	Dapk1	4.16	Gm5169	3.37
Gpr45	9.49	Gm3455	4.15	R3hcc1	3.37
D15Wsu169e	9.47	Gm14717	4.14	Slc38a1	3.37
Brunol5	9.44	1700001E04Rik	4.14	Inpp4b	3.37
Pxdn	9.44	Pde4a	4.13	Nphp3	3.37
Gpr83	9.43	Slc35f2	4.13	Csnk1g1	3.36
ENSMUSG00000072735	9.43	Adam6b	4.13	Jazf1	3.36
Gm3727	9.36	Penk	4.13	Arhgdig	3.36
Gm3727	9.25	2510048L02Rik	4.13	Etaa1	3.36
N/A	9.24	Casp3	4.12	Cul2	3.36
Gm11744	9.05	Dcaf17	4.12	Gm10837	3.36
Gm3339	8.66	Gm3182	4.12	Ppp2r3a	3.36
Dpy19l2	8.60	1500015O10Rik	4.11	Gm1574	3.35
Caskin2	8.31	Acsl4	4.11	Tspan12	3.35
Ikzf2	8.22	Ddx43	4.10	Magi3	3.35
Tubgcp5	8.17	AI987944	4.09	1110059M19Rik	3.35
Gm2974	8.16	Plin1	4.09	Cpsf4l	3.34
C230088H06Rik	8.08	Tox	4.09	Parp4	3.34
Fbxw27	8.05	Gm10338	4.07	Galr3	3.34
Gm14005	8.04	Zscan12	4.06	Adam33	3.34
Gm8362	7.95	Fam71e1	4.06	Frs3	3.33
Gm8297	7.93	Neb	4.06	Ptgdr	3.33
Pla2g2d	7.93	100039441	4.05	BE691133	3.33
Slc22a12	7.91	BC106179	4.05	Brp44l	3.33
N/A	7.87	N/A	4.05	Gm11468	3.33
Cadm3	7.81	Stab1	4.04	Dctn4	3.33
Cyhr1	7.58	Tnfsf13b	4.04	E330021D16Rik	3.33
B630019K06Rik	7.58	Mdfi	4.03	Gm3764	3.32
Inpp4b	7.50	A930002C04Rik	4.03	Cd300lg	3.32
Ctla4	7.49	Slc23a3	4.03	Atg2a	3.32
Cyp2u1	7.47	Col6a3	4.02	Ankrd9	3.32
Gm3182	7.44	Ghrh	4.01	Gm7225	3.32
Tgfb2	7.43	A930017M01Rik	4.01	Pnpla7	3.32
Vwce	7.41	Itih5l	4.01	Cd96	3.31
LOC100036568	7.32	Aurkc	4.00	4833422F24Rik	3.31
1700029I01Rik	7.31	Itga6	4.00	Thnsl2	3.31
Olfr701	7.29	Mfrp	3.99	Pdcd11	3.31
Rfc3	7.29	1700042G15Rik	3.99	Robo4	3.31
Gm10014	7.22	Mageh1	3.98	Aven	3.31
N/A	7.20	Ptpn13	3.98	1700026L06Rik	3.31
LOC100038847	7.16	Olfr227	3.98	Lrig2	3.31
544988	7.09	1700028M03Rik	3.98	Ehbp1	3.31
Gm4489	7.07	Gpatch4	3.98	Kctd9	3.30
LOC100038847	6.95	Pxmp2	3.97	Zbtb37	3.30
Nlrx1	6.94	Mllt3	3.97	Lrrc34	3.30
N/A	6.92	Gm10250	3.97	Zfp30	3.30
Gm3642	6.92	Cux1	3.96	Ano2	3.29
Tgm1	6.90	Csmd1	3.96	N/A	3.29
Dmd	6.88	Ptger3	3.96	Tmem134	3.29
Foxp3	6.85	Gm3990	3.95	Sh2d6	3.29
ENSMUSG00000072735	6.82	2010005J08Rik	3.94	Olfr78	3.29
Gria1	6.82	Olfr623	3.94	Mapk8	3.29
Arhgef15	6.81	ENSMUSG00000072735	3.94	Upp1	3.29
Gm2888	6.79	March7	3.94	Gm2046	3.29
Fdft1	6.73	N/A	3.94	Tex21	3.28
Gm3642	6.72	Slc9a3	3.93	Tnfrsf4	3.28
Nck2	6.70	Rbm9	3.93	Nol11	3.28
Adamtsl4	6.64	Dtwd1	3.93	1700092C10Rik	3.28
Zfp142	6.60	C77370	3.93	Gm3916	3.28
Gm3269	6.59	N/A	3.92	Dmxl2	3.28
Gm3411	6.56	Fbxw13	3.92	ENSMUSG00000072735	3.28
544988	6.53	Amz2	3.92	ENSMUSG00000079376	3.27
9630058J23Rik	6.53	Nsl1	3.92	4930587E11Rik	3.27
2010109N18Rik	6.51	Plxna3	3.92	Plcl1	3.27
N/A	6.51	Ppme1	3.90	Srgap3	3.27
Brap	6.51	Gcgr	3.90	Prss39	3.27
Tmem210	6.47	Sgcd	3.90	Dapk3	3.26
4930486G11Rik	6.46	N/A	3.89	Fbxw24	3.26
Vmn2r46	6.46	ENSMUSG00000068790	3.89	Gm3626	3.26
1110017D15Rik	6.46	Olfr658	3.88	Mtap4	3.26
N/A	6.43	Fbxo15	3.88	Gm3253	3.25
N/A	6.43	Mrgprb4	3.88	Cypt6	3.25
N/A	6.38	Ncoa7	3.87	Aatf	3.25
Gm3518	6.36	Grin1	3.87	Il2rb	3.25
C430002E04Rik	6.26	4933400A11Rik	3.87	Fam160a1	3.24
Gm13620	6.23	Vmn2r72	3.87	Ece1	3.24
Gm3685	6.23	Pfkp	3.87	Nkx2-6	3.24
Zscan10	6.22	Igl	3.86	Pik3r2	3.24
Gm10340	6.22	4930445K14Rik	3.86	Slc25a21	3.24
Gm3159	6.21	Krtap5-4	3.85	Ptgfrn	3.24
5830403L16Rik	6.16	Gm3424	3.85	N/A	3.24
Gm3127	6.15	Cd247	3.85	Tbcel	3.23
B930046C15Rik	6.13	Samsn1	3.85	Sgip1	3.23
Syne2	6.10	Uty	3.84	1700023L04Rik	3.23
Gm3029	6.10	Galk1	3.84	Catsper3	3.23
1600002D24Rik	6.06	1700029G01Rik	3.84	Dgka	3.23
Gm2224	6.04	Agrn	3.83	4930433N12Rik	3.23
Gm4801	6.00	Lrig2	3.83	Rdh16	3.22
N/A	5.97	Slc25a27	3.83	BC011248	3.22
Pitpnc1	5.94	Gjb4	3.83	Dlgap1	3.22
Gm3476	5.94	Rgs16	3.83	Olfr1283	3.22
6430562O15Rik	5.92	Cntn1	3.82	Osbpl3	3.22
Vmn2r66	5.92	Fndc7	3.82	Foxi2	3.21
Gm3029	5.91	Itk	3.82	Fam186a	3.20
Gm3115	5.89	N/A	3.82	Gm8356	3.20
Ndp	5.84	H1fx	3.81	Timp4	3.20
Zfp329	5.83	Pik3c2a	3.80	Tbc1d8	3.20
Gpr64	5.82	Ctsj	3.80	Srd5a1	3.20
Nav2	5.81	Emid1	3.80	Olfr242	3.20
Aven	5.80	Serpina1e	3.79	Sel1l	3.19
D030054H15Rik	5.79	Gm1330	3.79	Mbnl2	3.19
Grik5	5.76	Tbc1d4	3.79	Plac9	3.19
Sgsm3	5.71	Hs1bp3	3.79	N/A	3.19
Ovol2	5.70	Olfr961	3.79	Slc12a1	3.19
Mc1r	5.65	Pask	3.78	Zfp169	3.19
Gm10371	5.65	BC060267	3.78	Dok7	3.18
N/A	5.62	Kpna1	3.78	Gm2275	3.18
Luzp2	5.60	Arg1	3.77	Gm2643	3.18
Pthlh	5.59	Cts8	3.77	Dpep2	3.18
1700021F07Rik	5.58	Suclg1	3.77	Pard6b	3.18
Ccbe1	5.56	1700001E04Rik	3.77	Cyp17a1	3.17
Cul7	5.55	Synpo2	3.77	9330111N05Rik	3.17
Cpped1	5.55	6030458C11Rik	3.77	Ccdc33	3.17
Fmnl3	5.54	1190002H23Rik	3.77	Tub	3.17
D6Wsu163e	5.53	Rpusd3	3.76	Rpl7l1	3.17
Serpinb9d	5.49	Gm6710	3.76	AW495222	3.17
E030025P04Rik	5.49	Ikbkap	3.76	Ipcef1	3.17
Skap1	5.49	N/A	3.76	Tle2	3.17
D0H4S114	5.47	4921523L03Rik	3.75	B3galnt2	3.17
Piwil2	5.45	Speer4f	3.75	Ndel1	3.16
4930524L23Rik	5.45	Gm3047	3.75	Atp6v1c2	3.16
Ykt6	5.43	Synpr	3.74	Hnrpll	3.16
Slc24a3	5.40	N/A	3.73	Prrg1	3.16
N/A	5.39	9030624G23Rik	3.72	Cyp2j13	3.16
Gm6337	5.38	Trp53inp2	3.72	Espn	3.16
Gm3149	5.34	9130401M01Rik	3.72	Mup1	3.15
Tnfrsf9	5.29	Myst4	3.72	Ptprr	3.15
Ttn	5.28	Gm12836	3.72	Snx11	3.15
Gpr52	5.27	2810039B14Rik	3.71	Chchd8	3.15
Cntfr	5.26	Fastk	3.71	Dnm1	3.15
ENSMUSG00000079376	5.25	Inpp4b	3.71	Tbc1d25	3.15
LOC100038847	5.22	N/A	3.70	Olfr1120	3.14
Mybpc2	5.22	Prlh	3.70	Gm3981	3.14
Cdon	5.16	Mcm8	3.70	Morc2a	3.14
Slitrk6	5.16	Gm15340	3.70	Ttll7	3.14
Dom3z	5.14	Gm4926	3.69	Irf6	3.14
Gm3149	5.14	Ebpl	3.69	A830039H05Rik	3.13
Gm16521	5.14	N/A	3.69	1700024B18Rik	3.13
Smarcal1	5.14	4930417O13Rik	3.68	Trap1a	3.13
C230099D08Rik	5.13	Pcdh15	3.68	Vmn2r10	3.13
Olfr1252	5.11	Ctla4	3.68	Nrn1	3.13
4930599N23Rik	5.11	4933432I09Rik	3.68	Mapkapk3	3.13
Gm3642	5.10	Hsd17b2	3.68	3110082J24Rik	3.13
Gm5634	5.09	Fbp1	3.67	Ccdc65	3.13
Cngb1	5.08	Gm5795	3.66	Spag6	3.13
Pax3	5.07	Gm8159	3.66	AI428936	3.12
ENSMUSG00000068790	5.07	Atf7	3.66	Tiam1	3.12
4632404H12Rik	5.05	Kdm4a	3.66	Cenpk	3.12
Vill	5.03	Ocrl	3.66	Rapsn	3.12
Gm8050	5.02	Sgol1	3.65	Tm2d3	3.11
Anks4b	5.00	Prox2	3.65	Tiam1	3.11
St3gal6	4.96	Rnf26	3.65	Tle2	3.11
1700034I23Rik	4.96	N/A	3.64	Wbp11	3.11
Gm3172	4.95	Bub1	3.64	Olfr1128	3.11
Spata18	4.93	Trim63	3.63	Art1	3.11
Plcg1	4.92	Slc6a9	3.62	Grin3a	3.11
Has2as	4.91	Dst	3.61	1700110K17Rik	3.10
Ntn4	4.90	AI428936	3.61	Bcat1	3.10
Skap1	4.88	Marveld2	3.60	Iigp1	3.10
Cyp2c50	4.88	Esrrb	3.60	Pla2g4e	3.10
Cope	4.87	Gm4699	3.60	Rpusd1	3.10
N/A	4.86	Ttyh1	3.59	Olfr638	3.10
Gm3642	4.86	Rgs16	3.59	Agbl2	3.10
Tnfrsf18	4.85	8030463A06Rik	3.59	4921509O09Rik	3.09
N/A	4.82	4930578E11Rik	3.58	Olfr389	3.09
Snhg11	4.81	Cacnb2	3.57	Pcsk4	3.09
Gm6121	4.81	Setd3	3.57	Pou2f1	3.09
Ncoa7	4.80	N/A	3.57	Brcc3	3.09
1700025M24Rik	4.75	Gm2957	3.56	Gm3034	3.09
S100a7a	4.75	Magea3	3.56	Gm8362	3.09
Olfr140	4.74	Syngr3	3.56	D030028A08Rik	3.09
Crem	4.73	Gm3127	3.55	Fam118b	3.08
Gab3	4.72	ENSMUSG00000068790	3.55	Ccdc126	3.08
Ift80	4.71	Tmem176a	3.55	Fbxw4	3.08
Secisbp2	4.69	1700081N11Rik	3.55	Cish	3.08
1110019B22Rik	4.67	N/A	3.55	N/A	3.08
N/A	4.66	9530002K18Rik	3.54	A630023P12Rik	3.08
Gm7750	4.64	1700008F21Rik	3.54	Alox12b	3.07
N/A	4.61	Grhl3	3.54	Hsd3b4	3.07
N/A	4.61	Smc2	3.54	Caskin1	3.07
Gm8026	4.61	Fam46d	3.54	Ank3	3.07
4933407C03Rik	4.61	Mypop	3.54	Helz	3.06
Tmub2	4.59	Spats2	3.53	Taar7b	3.06
Tnfrsf25	4.59	Mpa2l	3.53	Gm3602	3.06
Gm3269	4.57	Nosip	3.53	Gm10094	3.06
Gm8297	4.57	Iigp1	3.53	Ptpn9	3.06
9130230L23Rik	4.56	Wdr52	3.51	1700085B03Rik	3.06
4831440E17Rik	4.55	4833442J19Rik	3.51	Gm7696	3.06
N/A	4.54	Tiam1	3.51	2610002I17Rik	3.06
Maf	4.54	Snapc4	3.51	Cav3	3.06
Gm7894	4.54	Dgat2	3.51	Slc4a8	3.06
4932431H17Rik	4.53	Saps2	3.50	Cacna2d1	3.06
E030046B03Rik	4.53	Tasp1	3.50	St3gal4	3.05
Gm3264	4.51	9930013L23Rik	3.50	Gm5134	3.05
Odz3	4.51	Sectm1b	3.49	Plod2	3.05
Olfr725	4.50	LOC432958	3.49	Gm2282	3.05
Frmd6	4.49	Grik2	3.49	Rpl26	3.05
Reck	4.47	B230216N24Rik	3.49	Ly6g6c	3.05
Cars2	4.47	Pla1a	3.49	Gm3453	3.05
Themis	4.46	Bex1	3.48	Suox	3.05
Msh2	4.46	N/A	3.48	Emilin3	3.05
Olfr1356	4.45	Slc35d1	3.48	4931422A03Rik	3.05
E030010N08Rik	4.44	N/A	3.47	Airn	3.05
Ninj2	4.44	Zfp444	3.47	Gm8301	3.04
Dennd2c	4.44	Kcnab3	3.47	Prss23	3.04
LOC100038847	4.43	Gm9893	3.47	Exoc3l	3.04
Ppp2r3a	4.42	Afm	3.46	Gm3556	3.04
Rsad1	4.42	Tecpr1	3.46	Car12	3.04
Nicn1	4.41	Gm7980	3.46	N/A	3.04
N/A	4.40	V1rc26	3.46	Ipcef1	3.03
Osbpl3	4.38	Pyroxd2	3.46	Gm6160	3.03
Duxbl	4.38	Myo1b	3.45	Stk30	3.03
Olfr1019	4.38	Gemin5	3.45	Txk	3.03
Ripk4	4.37	Dzip1	3.45	Klra4	3.03
Ermp1	4.37	Pabpc3	3.45	Icos	3.03
Sfmbt2	4.33	Olfr781	3.45	Ciapin1	3.02
Gpt2	4.33	Agrn	3.44	Frmd4b	3.02
Myct1	4.32	Fam98c	3.44	Gm3278	3.02
E330026B02Rik	4.31	Fam65a	3.44	Scrn3	3.02
Zbtb16	4.31	Plekhg1	3.44	0610031O16Rik	3.02
N/A	4.29	Pbld	3.44	Brwd2	3.02
2010005H15Rik	4.29	Epb4.1l1	3.43	Numbl	3.02
Rragd	4.28	Zap70	3.43	Raph1	3.02
Ephb3	4.28	Kcnk13	3.43	N/A	3.01
Treh	4.28	Mrgprh	3.43	N/A	3.01
Krt72	4.28	Gm8519	3.42	Klrg1	3.01
Snx16	4.28	Cntfr	3.42	Srd5a1	3.01
Tox	4.28	N/A	3.41		

**Figure 3:**
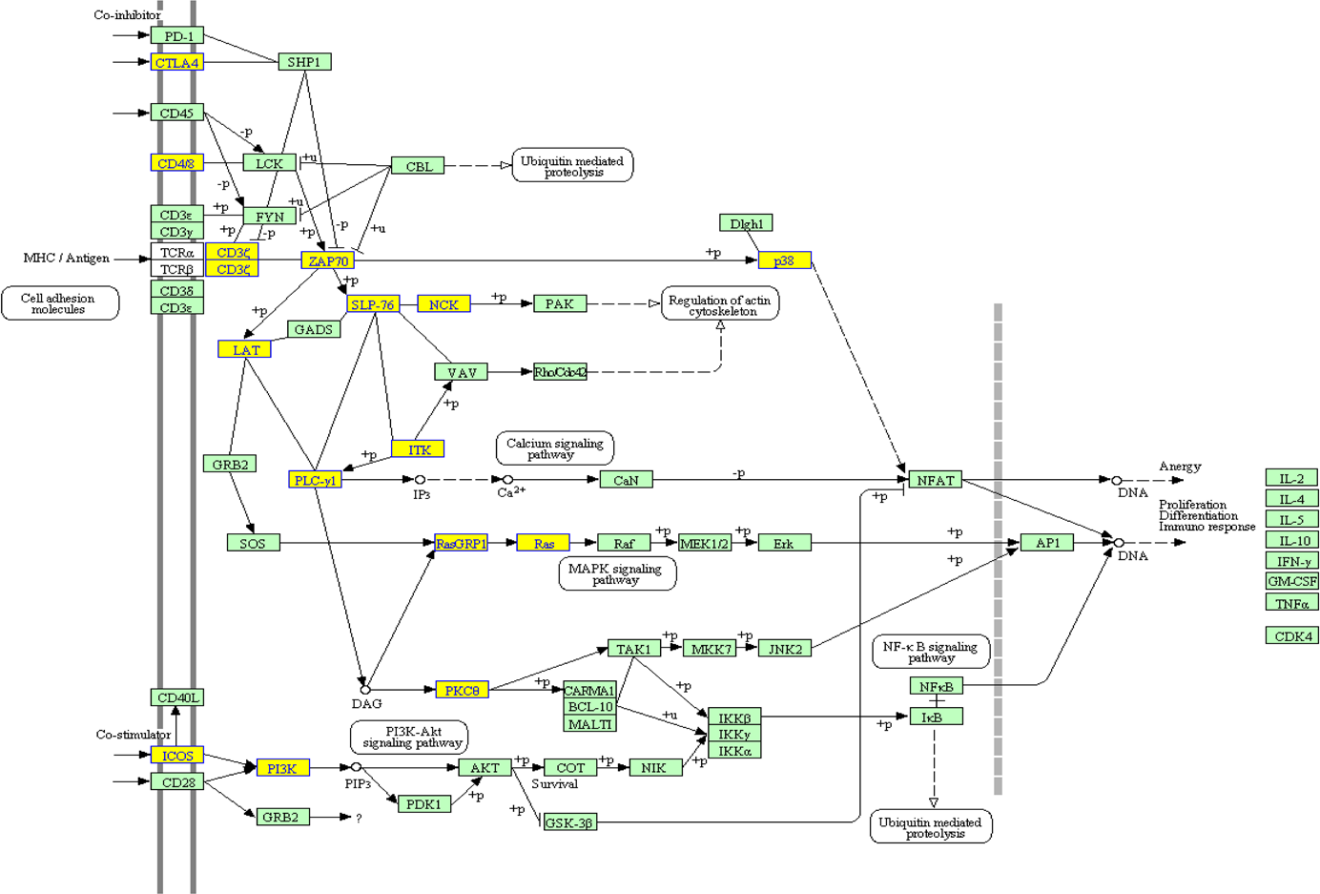
Abnormal target genes of differentially expressed miRNAs were significantly enriched in the TCR signaling pathway. The *p* value calculated by the hypergeometric distribution was set to 0.01. Downexpressed genes were shown in yellow.

To clarify which signaling pathways were altered in CCR6^+^ Tregs, we applied the KEGG library and performed enrichment analysis for microarray data. Twenty signaling pathways were enriched with the criteria of 2 fold changes (Table 4), which include the inositol phosphate metabolism, T cell receptor signaling pathway, phosphatidylinositol signaling system, mTOR signaling pathway, primary immunodeficiency and some cancer signaling pathway. Some genes from those pathways were downregulated or upregulated, such as in T cell receptor signaling pathway, ICOS, ZAP70, LAT, PLC-*γ*1, ITK, Ras and p38 were downregulated (Fig. 3). The mTOR pathway evenly consisted of both up and downregulated genes, of which RSK, STRAD and Raptor were downregulated and PIK3c2b, TSC1 and MO25 were upregulated (Tables 2 and 3).

**Table 4:** KEGG pathways annotation of abnormal miRNA target genes.

Pathway	MAPP name	Enrichment Score
mmu00562	Inositol phosphate metabolism	3.988221
mmu04070	Phosphatidylinositol signaling system	3.533671
mmu05410	Hypertrophic cardiomyopathy (HCM)	2.394271
mmu04725	Cholinergic synapse	2.227839
mmu05412	Arrhythmogenic right ventricular cardiomyopathy (ARVC)	2.126784
mmu04724	Glutamatergic synapse	2.109772
mmu03460	Fanconi anemia pathway	2.017738
mmu05142	Chagas disease (American trypanosomiasis)	2.010757
mmu04150	mTOR signaling pathway	1.906663
mmu04660	T cell receptor signaling pathway	1.713143
mmu05322	Systemic lupus erythematosus	12.6937
mmu04640	Hematopoietic cell lineage	6.723747
mmu05034	Alcoholism	6.20107
mmu05152	Tuberculosis	5.152889
mmu04662	B cell receptor signaling pathway	4.675411
mmu05202	Transcriptional misregulation in cancer	4.643977
mmu04672	Intestinal immune network for IgA production	4.281526
mmu04380	Osteoclast differentiation	4.255375
mmu05150	Staphylococcus aureus infection	3.867061
mmu05340	Primary immunodeficiency	3.857659

**Notes.**

a Gray indicates downregulated target genes in the KEGG pathway.

b In differentially expressed genes, 15 miRNA target genes were enriched into T cell receptor (TCR) signaling pathway (Fig. 3).

## Discussion

Previous studies have indicated that CD4^+^CD25^+^ regulatory T cells (Treg) were a heterogeneous cell population comprising different subsets that play distinct roles in diverse animal models or human clinical disease, mediating immune suppression or immune tolerance (Pankratz et al., 2014; Erfani et al., 2014; Lee et al., 2014). Thus, the investigation involved in regulation of function of distinct subset of Tregs is valuable. Recent evidence showed that CCR6^+^ subsets of Tregs played an important role in various immune responses such as Villares et al.’s (2009) report that CCR6^+^ Tregs could inhibit the function of pathological CD4^+^ Th1 cells mediated EAE pathology. We also found that CCR6^+^ Tregs, but not their CCR6^-^ counterpart, could dominantly enrich in tumor mass and potential inhibited the function of effector T cells *in vivo* (Xu et al., 2010; Xu et al., 2011). These findings might support the fact that CCR6^+^ subset of Tregs played a critical role in tumor immunity. However, the regulation mechanism involved in the function of this subset Tregs remains largely unknown. Recent studies provided some clues to this problem since they showed that miRNAs may play a regulatory role in the development and function of Tregs (Smigielska-Czepiel et al., 2014; Fayyad-Kazan et al., 2012). To gain new insight into the role of miRNAs in the function of CCR6^+^ Tregs, differentially expressed miRNAs were investigated by microarray assay. Moreover, the regulatory pathways of putative target genes were also analyzed in CCR6^+^ Tregs. It was found that there were significantly different miRNA expression patterns in CCR6^+^ Tregs and CCR6^-^ Tregs. The difference could describe one handred and twenty miRNAs, including 58 up- and 62 down-regulated, which had more than 2-fold differential expression between CCR6^+^ Tregs and CCR6^-^ Tregs. We speculated that the above two differences might be a reason for different functions such as proliferation activity of CCR6^+^ Tregs compared with CCR6^-^ Tregs.

miR-142, a distinct member of the miRNA family, is highly conserved across species and is linked to chromosome 3p22.3/12q14. Recent evidence showed that miR-142 was highly expressed in Tregs and could regulate the expansion of Tregs in response to stimulation (Zhou et al., 2013). In this study, we observed that miR-142 was significantly upregulated in CCR6^+^ Tregs. Predicated by TargetScan and FINDTAR3, 14 genes were putative targets of miR-142, in which 4 genes was downregulated (Fig. S2). We also noticed that Gfi1 was downregulated in CCR6^+^ Tregs, indicating that Gfi1 might be a target of miR-142. Shi et al. (2013a) and Shi et al. (2013b) demonstrated that Gfi1 was critical for the development of Tregs. Moreover, loss of Gfi-1 could endow the aberrant expansion of Tregs through IL-2 signaling pathway. Thus, further study on miR-142 function will help us understand the regulatory role of miR-142 in the function and proliferation of CCR6^+^ Tregs.

MiR-21 is one of the first discovered miRNAs that is known to be widespread in human tissues such as heart, lung, brain and liver. Further data has highlighted that miR-21 can regulate the biological character of various cells including survival, invasion and apoptosis (Shi et al., 2013a; Shi et al., 2013b; Bullock et al., 2013; Niu et al., 2012). In particular miR-21 was documented as an important regulator actor in the proliferation of cells. For example, Liu et al. (2014) reported that miR-21 could enhance the proliferation of cancer cells through the Akt pathway. Interestingly, recent evidence further showed that miR-21 was also functionally expressed in T cells (Sommers et al., 2013), miR-21 could support survival of CD4^+^ T cells, and was an important signature in CD4^+^ T cells proliferation. Also, silencing of miR-21 could alter the proportion of CD4^+^ T cells in lupus mice (Wang et al., 2014). Consistently, we observed an increase in the expression of miR-21 in CCR6^+^ Tregs. Therefore, further study on the possible role of miR-21 was also valuable for the understanding of proliferation of CCR6^+^ Tregs.

The data from gene expression microarray showed that 1,391 genes (651 downregulated and 740 up-regulated) significantly changed more than three fold in CCR6^+^ Tregs. Among them, some genes have been demonstrated to be involved in the proliferation and function of Tregs. For example, TCR signaling pathway was critical for the proliferation and function of CCR6^+^ Tregs. We noticed that some genes, including ZAP70, LAT and PLC-1 were downregulated, indicating weak transduction of TCR signaling pathway in CCR6^+^ Tregs. Consistently, previous literature showed that CCR6^+^ Tregs demonstrated a memory/effector phenotype and more sensitivity for the stimulation of TCR (Kleinewietfeld et al., 2005). In addition, Hanschen et al. (2012) reported that TCR stimulation could induce rapid and higher activation of ZAP70 in Tregs, indicating that phosphorylation of ZAP70 also might be important for the proliferation of CCR6^+^ Tregs. Therefore, these altered genes might be good targets for the proliferation and function of CCR6^+^ Tregs in successive research work. In addition, we would point out that we did not find any prominently annotated biological category using miRNA-mRNA anti-correlations in the present study. We propose this reflects the fact that the interaction of miRNA and mRNA in the biology of CCR6^+^ Tregs is complex which remains to be elucidated in future work.

In summary, to our knowledge, this is the first time that CCR6^+^ Tregs, a distinct subset of Tregs, has been shown to express a distinct miRNA profile; this will help us to understand the potential role of miRNAs in the biological function of CCR6^+^ Tregs.

## Supplemental Information

10.7717/peerj.575/supp-1 Figure S1 The relative expression of miR-142 and miR-21 in CCR6^+^ Tregs CCR6^+^ Tregsand CCR6^-^ Tregs were purified from splenocytes in Balb/c mice by FACSsorting. The relative expression of miR-142 and miR-21 in CCR6^+^ Tregscells was determined by Realtime PCR assay.

10.7717/peerj.575/supp-2 Figure S2 The expression of putative targets of miR-142a in CCR6^+^ Tregs CCR6^+^ Tregsand CCR6^-^ Tregs were purified from splenocytes in Balb/c mice by FACSsorting. The global expression of genes in cells was analyzed by microarrayarray and then the expression of putative target genes was listed (CCR6^-^ Tregs; CCR6^+^ Tregs)

## Additional Information and Declarations

### Microarray Data Deposition

The following information was supplied regarding the deposition of microarray data: NCBI GEO database accession: GSE60041.

## References

[ref-1] Bullock MD, Pickard KM, Nielsen BS, Sayan AE, Jenei V, Mellone M, Mitter R, Primrose JN, Thomas GJ, Packham GK, Mirnezami AH (2013). Pleiotropic actions of miR-21 highlight the critical role of deregulated stromal microRNAs during colorectal cancer progression. Cell Death & Disease.

[ref-2] Chen KJ, Lin SZ, Zhou L, Xie HY, Zhou WH, Taki-Eldin A, Zheng SS (2011). Selective recruitment of regulatory T cell through CCR6^-^ CCL20 in hepatocellular carcinoma fosters tumor progression and predicts poor prognosis. PLoS ONE.

[ref-3] Danger R, Braza F, Giral M, Soulillou JP, Brouard S (2014). MicroRNAs, major players in B cells homeostasis and function. Frontiers in Immunology.

[ref-4] Duhen T, Campbell DJ (2014). IL-1*β* promotes the differentiation of polyfunctional human CCR6^+^ CXCR3^+^ Th1/17 cells that are specific for pathogenic and commensal microbes. Journal of Immunology.

[ref-5] Erfani N, Hamedi-Shahraki M, Rezaeifard S, Haghshenas M, Rasouli M, Samsami Dehaghani A (2014). Foxp3^+^ T cells in peripheral blood of patients with epithelial ovarian cancer. Iranian Journal of Immunology.

[ref-6] Fayyad-Kazan H, Rouas R, Fayyad-Kazan M, Badran R, El Zein N, Lewalle P, Najar M, Hamade E, Jebbawi F, Merimi M, Romero P, Burny A, Badran B, Martiat P (2012). MicroRNA profile of circulating CD4-positive regulatory T cells in human adults and impact of differentially expressed microRNAs on expression of two genes essential to their function. Journal of Biological Chemistry.

[ref-7] Gigli I, Maizon DO (2013). microRNAs and the mammary gland: a new understanding of gene expression. Genetics and Molecular Biology.

[ref-8] Hanschen M, Tajima G, O’Leary F, Hoang K, Ikeda K, Lederer JA (2012). Phospho-flow cytometry based analysis of differences in T cell receptor signaling between regulatory T cells and CD4^+^ T cells. Journal of Immunological Methods.

[ref-9] Johanson TM, Skinner JP, Kumar A, Zhan Y, Lew AM, Chong MM (2014). The role of microRNAs in lymphopoiesis. International Journal of Hematology.

[ref-10] Kallal LE, Schaller MA, Lindell DM, Lira SA, Lukacs NW (2010). CCL20/CCR6 blockade enhances immunity to RSV by impairing recruitment of DC. European Journal of Immunology.

[ref-11] Kitamura K, Farber JM, Kelsall BL (2010). CCR6 marks regulatory T cells as a colon-tropic, IL-10–producing phenotype. Journal of Immunology.

[ref-12] Kleinewietfeld M, Puentes F, Borsellino G, Battistini L, Rötzschke O, Falk K (2005). CCR6 expression defines regulatory effector/memory-like cells within the CD25^+^ CD4^+^ T-cell subset. Blood.

[ref-13] Lamprecht B, Kreher S, Anagnostopoulos I, Jöhrens K, Monteleone G, Jundt F, Stein H, Janz M, Dörken B, Mathas S (2008). Aberrant expression of the Th2 cytokine IL-21 in Hodgkin lymphoma cells regulates STAT3 signaling and attracts Treg cells via regulation of MIP-3*α*. Blood.

[ref-14] Lee KM, Stott RT, Zhao G, SooHoo J, Xiong W, Lian MM, Fitzgerald L, Shi S, Akrawi E, Lei J, Deng S, Yeh H, Markmann JF, Kim JI (2014). TGF-*β*-producing regulatory B cells induce regulatory T cells and promote transplantation tolerance. European Journal of Immunology.

[ref-15] Liu Y, Nie H, Zhang K, Ma D, Yang G, Zheng Z, Liu K, Yu B, Zhai C, Yang S (2014). A feedback regulatory loop between HIF-1*α* and miR-21 in response to hypoxia in cardiomyocytes. FEBS Letters.

[ref-16] Niu J, Shi Y, Tan G, Yang CH, Fan M, Pfeffer LM, Wu ZH (2012). DNA damage induces NF-*κ* B-dependent microRNA-21 up-regulation and promotes breast cancer cell invasion. Journal of Biological Chemistry.

[ref-17] Pankratz S, Bittner S, Herrmann AM, Schuhmann MK, Ruck T, Meuth SG, Wiendl H (2014). Human CD4^+^ HLA-G^+^ regulatory T cells are potent suppressors of graft-versus-host disease *in vivo*. The FASEB Journal.

[ref-18] Paradis M, Mindt BC, Duerr CU, Rojas OL, Ng D, Boulianne B, McCarthy DD, Yu MD, Summers deLuca LE, Ward LA, Waldron JB, Philpott DJ, Gommerman JL, Fritz JH (2014). A TNF-*α*–CCL20–CCR6 axis regulates nod1-induced B Cell Responses. Journal of Immunology.

[ref-19] Rivino L, Gruarin P, Häringer B, Steinfelder S, Lozza L, Steckel B, Weick A, Sugliano E, Jarrossay D, Kühl AA, Loddenkemper C, Abrignani S, Sallusto F, Lanzavecchia A, Geginat J (2010). CCR6 is expressed on an IL-10–producing, autoreactive memory T cell population with context-dependent regulatory function. Journal of Experimental Medicine.

[ref-20] Shi LZ, Kalupahana NS, Turnis ME, Neale G, Hock H, Vignali DA, Chi H (2013a). Inhibitory role of the transcription repressor Gfi1 in the generation of thymus-derived regulatory T cells. Proceedings of the National Academy of Sciences of the United States of America.

[ref-21] Shi Z, Zhang J, Qian X, Han L, Zhang K, Chen L, Liu J, Ren Y, Yang M, Zhang A, Pu P, Kang C (2013b). AC1MMYR2, an inhibitor of dicer-mediated biogenesis of oncomir miR-21, reverses epithelial–mesenchymal transition and suppresses tumor growth and progression. Cancer Research.

[ref-22] Smigielska-Czepiel K, Van den Berg A, Jellema P, Van der Lei RJ, Bijzet J, Kluiver J, Boots AM, Brouwer E, Kroesen BJ (2014). Comprehensive analysis of miRNA expression in T-cell subsets of rheumatoid arthritis patients reveals defined signatures of naive and memory Tregs. Genes & Immunity.

[ref-23] Sommers CL, Rouquette-Jazdanian AK, Robles AI, Kortum RL, Merrill RK, Li W, Nath N, Wohlfert E, Sixt KM, Belkaid Y, Samelson LE (2013). miRNA signature of mouse helper T cell hyper-proliferation. PLoS ONE.

[ref-24] Villares R, Cadenas V, Lozano M, Almonacid L, Zaballos A, Martínez-A C, Varona R (2009). CCR6 regulates EAE pathogenesis by controlling regulatory CD4^+^ T-cell recruitment to target tissues. European Journal of Immunology.

[ref-25] Wang L, He L, Zhang R, Liu X, Ren Y, Liu Z, Zhang X, Cheng W, Hua ZC (2014). Regulation of T lymphocyte activation by microRNA-21. Molecular Immunology.

[ref-26] Wong EB, Akilimali NA, Govender P, Sullivan ZA, Cosgrove C, Pillay M, Lewinsohn DM, Bishai WR, Walker BD, Ndung’u T, Klenerman P, Kasprowicz VO (2013). Low levels of peripheral CD161^+^ CD8^+^ mucosal associated invariant T (MAIT) cells are found in HIV and HIV/TB co-infection. PLoS ONE.

[ref-27] Xu L, Xu W, Qiu S, Xiong S (2010). Enrichment of CCR6Foxp3 regulatory T cells in the tumor mass correlates with impaired CD8 T cell function and poor prognosis of breast cancer. Clinical Immunology.

[ref-28] Xu L, Xu W, Wen Z, Xiong S (2011). *In situ* prior proliferation of CD4^+^ CCR6^+^ regulatory T cells facilitated by TGF-*β* secreting DCs is crucial for their enrichment and suppression in tumor immunity. PLoS ONE.

[ref-29] Zhou Q, Haupt S, Prots I, Thümmler K, Kremmer E, Lipsky PE, Schulze-Koops H, Skapenko A (2013). miR-142-3p is involved in CD25^+^ CD4 T cell proliferation by targeting the expression of glycoprotein A repetitions predominant. Journal of Immunology.

