# Supplementary material for: MicroRNAs expression profile in CCR6+ regulatory T cells
Source: PeerJ. 2014 Sep 18;2:e575. doi: 10.7717/peerj.575 (PMC4179613; doi:10.7717/peerj.575)
Supplement: Figure S1 — CCR6+ Tregsand CCR6- Tregs were purified from splenocytes in Balb/c mice by FACSsorting. The relative expression of miR-142 and miR-21 in CCR6+ Tregscells was determined by Realtime PCR assay. [file peerj-02-575-s001.docx]

**
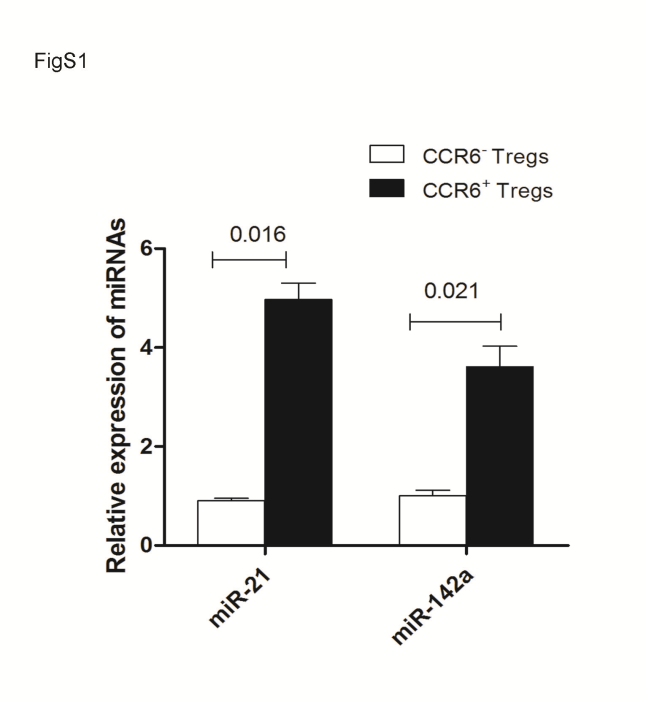
**

**Figure S1. The relative expression of miR-142 and miR-21 in CCR6^+^Tregs.**

CCR6^+^Tregs and CCR6^-^Tregs were purified from splenocytes in Balb/c mice by FACS sorting. The relative expression of miR-142 and miR-21 in CCR6^+^Tregs cells was determined by Realtime PCR assay.
