# Supplementary material for: MicroRNAs expression profile in CCR6+ regulatory T cells
Source: PeerJ. 2014 Sep 18;2:e575. doi: 10.7717/peerj.575 (PMC4179613; doi:10.7717/peerj.575)
Supplement: Figure S2 — CCR6+ Tregsand CCR6- Tregs were purified from splenocytes in Balb/c mice by FACSsorting. The global expression of genes in cells was analyzed by microarrayarray and then the expression of putative target genes was listed (CCR6- Tregs; CCR6+ Tregs) [file peerj-02-575-s002.docx]

**
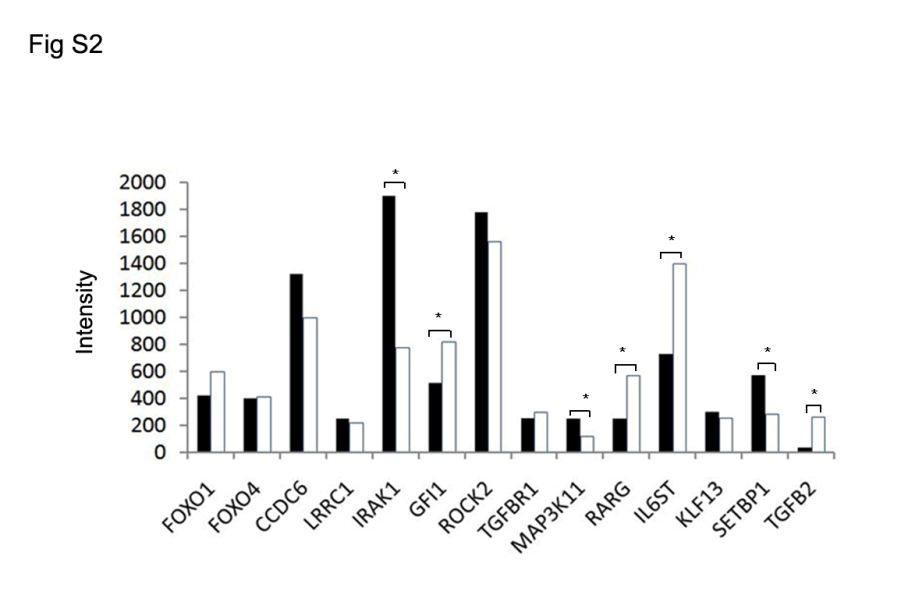
**

**Figure S2.** The expression of putative targets of miR-142a in CCR6^+^Tregs**.**

CCR6^+^Tregs and CCR6^-^Tregs were purified from splenocytes in Balb/c mice by FACS sorting. The global expression of genes in cells was analyzed by microarray array and then the expression of putative target genes was listed (. CCR6^-^Tregs; CCR6^+^Tregs)
